# Distinct Kinin-Induced Functions Are Altered in Circulating Cells of Young Type 1 Diabetic Patients

**DOI:** 10.1371/journal.pone.0011146

**Published:** 2010-06-17

**Authors:** Nicolle Kränkel, Stephen Paul Armstrong, Craig Alexander McArdle, Colin Dayan, Paolo Madeddu

**Affiliations:** 1 Experimental Cardiovascular Medicine, University of Bristol, Bristol, United Kingdom; 2 Laboratories for Integrated Neuroscience and Endocrinology, University of Bristol, Bristol, United Kingdom; Universität Würzburg, Germany

## Abstract

**Aims/Hypothesis:**

We aimed to understand early alterations in kinin-mediated migration of circulating angio-supportive cells and dysfunction of kinin-sensitive cells in type-1 diabetic (T1D) patients before the onset of cardiovascular disease.

**Methods:**

Total mononuclear cells (MNC) were isolated from peripheral blood of 28 T1D patients free from cardiovascular complications except mild background retinopathy (age: 34.8±1.6 years, HbA_1C_: 7.9±0.2%) and 28 age- and sex-matched non-diabetic controls (H). We tested expression of kinin receptors by flow cytometry and migratory capacity of circulating monocytes and progenitor cells towards bradykinin (BK) in transwell migration assays. MNC migrating towards BK (BK^mig^) were assessed for capacity to support endothelial cell function in a matrigel assay, as well as generation of nitric oxide (NO) and superoxide (O_2_^−^*) by using the fluorescent probes diaminofluorescein and dihydroethidium.

**Results:**

CD14^hi^CD16^neg^, CD14^hi^CD16^pos^ and CD14^lo^CD16^pos^ monocytes and circulating CD34^pos^ progenitor cells did not differ between T1D and H subjects in their kinin receptor expression and migration towards BK. T1D BK^mig^ failed to generate NO upon BK stimulation and supported endothelial cell network formation less efficiently than H BK^mig^. In contrast, O_2_^−^* production was similar between groups. High glucose disturbed BK-induced NO generation by MNC-derived cultured angiogenic cells.

**Conclusions/Interpretation:**

Our data point out alterations in kinin-mediated functions of circulating MNC from T1D patients, occurring before manifest macrovascular damage or progressed microvascular disease. Functional defects of MNC recruited to the vessel wall might compromise endothelial maintenance, initially without actively promoting endothelial damage, but rather by lacking supportive contribution to endothelial regeneration and healing.

## Introduction

In patients with diabetes mellitus, vascular function deteriorates faster and cardiovascular complications occur more frequently than in the non-diabetic population. Enhanced and continuous recruitment of circulating inflammatory cells characterizes developing atherosclerotic lesions. At the same time, circulating progenitor cells (CPC) and distinct monocyte subtypes - which are able to support endothelial homeostasis, modulate inflammation and mediate repair - become dysfunctional and their recruitment is disturbed [1]–[3].

Although diabetes-associated alterations, like enhanced glycoxidative stress and insulin deficiency, directly affect endothelial cell (EC) survival and function, recruited cells have a critical role in further modulating vascular function by secretion of cytokines, proteases and radicals, like nitric oxide (NO) or superoxide (O_2_^−^*). Reduced availability of NO, important for angiogenesis and maintenance of endothelial integrity, together with increased generation of O_2_^−^*, a marker of inflammation and mediator of atherosclerosis, are implicated in decline of vascular function in diabetes [4]–[7]. Distinct types of recruited cells can generate differential amounts of NO and O_2_^−^*, depending on their specific processing of stimuli, as well as pathology-induced dysfunction.

In the vessel wall, one of the mechanisms generating NO and O_2_^−^* is the kallikrein-kinin-system (KKS). The KKS regulates a variety of (patho-)physiological processes, including vascular homeostasis, inflammation, angiogenesis, coagulation and vessel wall remodeling [8], [9]. Kinins, the effectors of the KKS, signal through G-protein coupled receptors, most prominently the constitutive B2R and the inducible B1R [9]. Both receptors differ with regard to their cell type-specific expression, dynamics of subcellular localization, and downstream signaling, thus introducing various levels of regulation. According to current understanding, the B1R affects inflammatory responses, while the B2R mediates vasorelaxation, endothelial homeostasis and angiogenesis [8], [9]. However, recent observations indicate a more complex role of both receptors in cardiovascular pathologies, which still need to be further elucidated [10].

We have recently demonstrated the importance of the B2R in the recruitment of circulating pro-angiogenic cell types as well as in the subsequent mounting of revascularization and recovery of blood flow in ischemic tissue [11]. Furthermore, the B2R ligand bradykinin (BK) is able to induce NO generation in resident EC, as well as O_2_^−^*, depending on the (patho-)physiological context [9], [12]–[14]. Deregulation of kinin signaling in diabetes might therefore underlie the observed alterations in recruitment of circulating cells, as well as paracrine effects of recruited cells upon the endothelium, e.g. via generation of O_2_^−^* rather than NO.

In the present study, we investigate alterations in kinin receptor expression on angio-supportive circulating cell types, namely CD34^pos^ CPC and monocytes, and kinin-induced cellular functions, such as migration and generation of O_2_−* and NO, in type 1 diabetic patients (T1D) prior to the onset of cardiovascular disease. Results indicate the presence of functional alterations in circulating MNC which does not affect their homing in response to kinins, but may render them less efficient in supporting endothelial homeostasis by paracrine ways well before clinical manifestation of cardiovascular complications.

## Results

### Patients' characteristics

T1D and H subjects did not differ with regard to factors influencing cardiovascular risk *(* ***Table 1*** *)*. All T1D patients were free from cardiovascular complications as ascertained by clinical evaluation, except for the presence of mild background retinopathy according to the scale of the UK national screening committee for diabetic retinopathy. Furthermore, HbA_1C_ was significantly higher in T1D as compared to H (***Table 1***). As the amount of blood available was limited due to restrictions imposed by the local ethics committee, assays were performed on subgroups. No difference regarding cardiovascular risk factors was found between T1D and H subjects when analyzing for subgroups, while HbA_1C_ was still significantly elevated in T1D (*data not shown*).

**Table 1:** Characteristics of the study populations.

	*H*	*T1D*
***N***	28	28
***Gender*** * [% male]*	50%	50%
***Mean age*** * [years]*	32.3±1.7	34.8±1.6
***Mean duration of T1D*** * [years]*	-	21.9±1.4
***background retinopathy*** * [% of patients]*	0	100
***CAD*** * [% of patients]*	0	0
***PAD*** * [% of patients]*	0	0
***Mean HbA1C [%]***	5.3±0.1	7.9±0.2***
***Medication*** * [% of patients]*		
***Insulin***	0%	100%
***ACE-inhibitors***	0%	0%
***Statins***	0%	6.7%
***AngII receptor blocker***	0%	0%

Values are mean±SEM (where applicable).

*** P<0.001 vs. H.

### Availability and characterization of circulating progenitor cells (CPC) and monocytes

**Figure 1:**
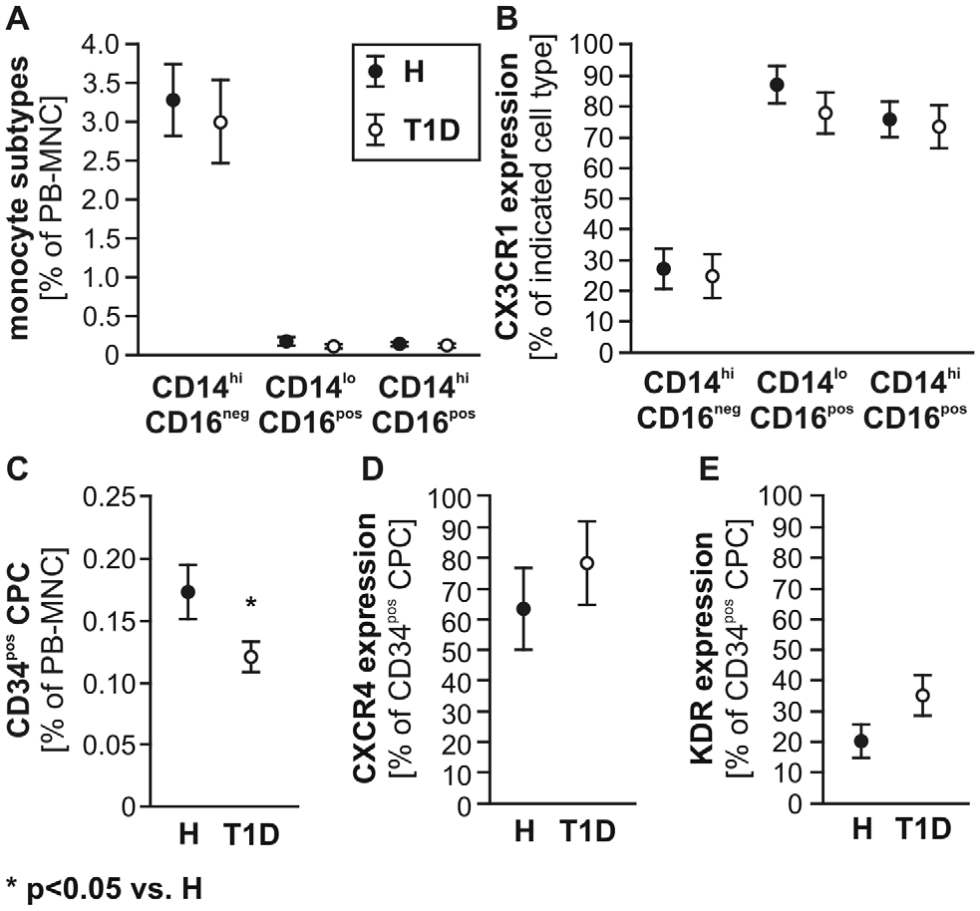
Characterization of monocyte and CPC in peripheral blood. No significant alterations were detected in the percentage of monocyte subtypes between non-diabetic (H, full circles) and T1D (empty circles) donors (**A**). CX3CR1 was mainly expressed by CD16^pos^ monocyte subtypes, with no difference between study groups (**B**). CD34^pos^ CPC percentage among PB-MNC was lower in T1D patients (**C**, * P<0.05 vs. H), while co-expression of CXCR4 (**D**) and KDR (**E**) was slightly, but not significantly increased on CD34^pos^ CPC of T1D patients as compared to H subjects. Values are mean±SEM, n = 14 (monocytes in **A & B**) and n = 20 (CPC in **C–E**) per group.

Monocyte and lymphocyte counts did not differ between H and T1D in the whole study groups (***Table 2***) or in subgroups (*data not shown*). Numbers of CD14^hi^CD16^neg^ “classical” monocytes, as well as CD14^hi^CD16^pos^ and CD14^lo^CD16^pos^ inflammatory/regulatory monocytes in blood did not differ between H and T1D donors (***Fig. 1A***). The fractalkine receptor CX3CR1, previously associated with recruitment of pro-atherosclerotic cells and their retention in the vessel wall [15], was co-expressed on the majority of CD16^pos^ monocyte subtypes and less frequent on CD14^hi^CD16^neg^ monocytes, with no significant differences between H and T1D (***Fig. 1B***). In contrast, percentage of CD34^pos^ CPC was lower in the T1D as compared to the H group *(* ***Fig. 1C*** *)*. Co-expression of KDR or CXCR4, previously associated with homing capacity of circulating endothelial progenitor cells, was slightly, but not significantly higher in T1D as compared to CD34^pos^ CPC from H subjects (***Fig. 1D&E***).

**Table 2:** Blood cell count of patient groups.

	*H*	*T1D*
***erythrocytes*** * [n per mL PB]*	4.8×10^9^±8.7×10^7^	4.8×10^9^±8.0×10^7^
***lymphocytes*** * [n per mL PB]*	1.8×10^6^±1.1×10^5^	1.9×10^6^±1.1×10^5^
***monocytes*** * [n per mL PB]*	4.4×10^5^±3.2×10^4^	4.2×10^5^±2.8×10^5^
***neutrophils*** * [n per mL PB]*	3.4×10^6^±2.4×10^5^	3.2×10^6^±1.8×10^5^
***eosinophils*** * [n per mL PB]*	1.0×10^5^±1.3×10^4^	1.9×10^5^±2.2×10^4^ ***
***basophils*** * [n per mL PB]*	3.1×10^4^±3.2×10^3^	3.1×10^4^±3.2×10^3^

Values are mean±SEM.

*** P<0.001 vs. H.

### Kinin receptor expression on circulating cells

**Figure 2:**
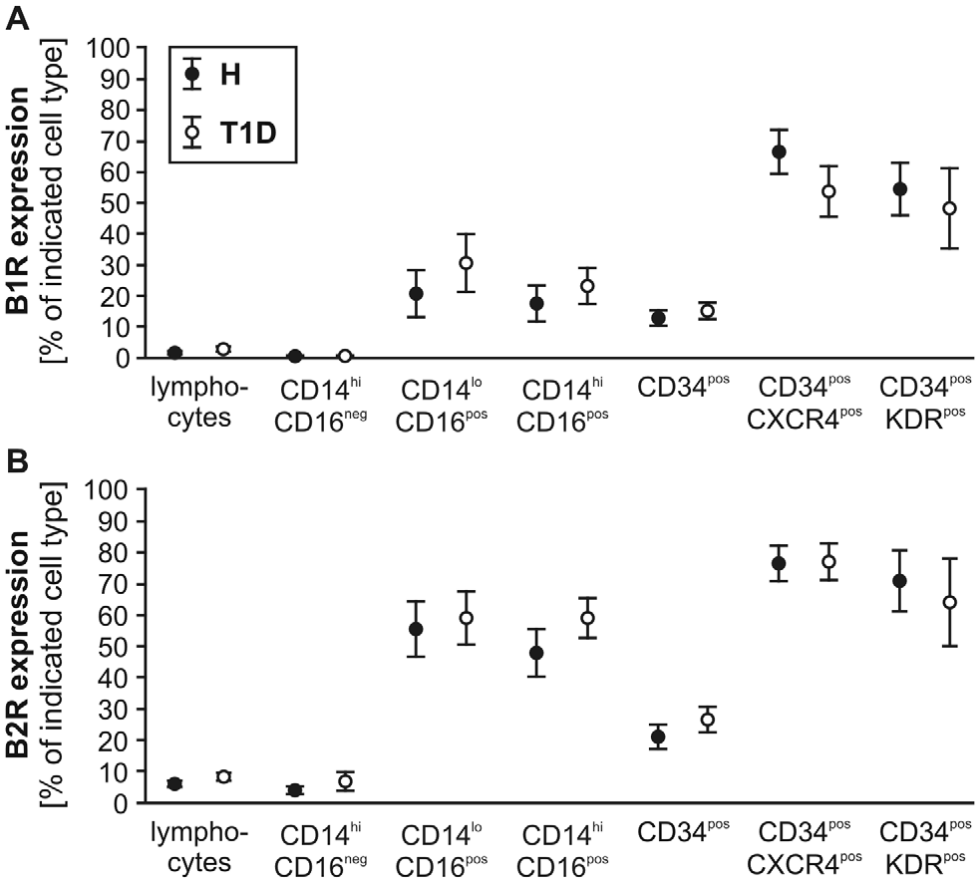
Kinin receptor expression on distinct cell types in peripheral blood. Both kinin receptors were mainly expressed on CD16^pos^ monocytes and KDR or CXCR4 co-expressing CPC, but no significant changes in the co-expression of the B1R (**A**) or the B2R (**B**) were detected on monocytes or CPC of T1D vs. H donors. Values are mean±SEM, n = 14 (monocytes), n = 20 (CPC) and n = 17 (lymphocytes) per group.

Kinin receptor expression was low on lymphocytes and CD14^hi^CD16^neg^ monocytes *(* ***Fig. 2*** *)*. In contrast, both kinin receptors were expressed more frequently on CD16^pos^ monocyte subtypes and CD34^pos^ CPC co-expressing KDR or CXCR4, with a prevalence of the B2R over the B1R *(* ***Fig. 2*** *)*. No differences in the expression of kinin receptors were detected between patient groups. Likewise, the ratio of kinin receptor subtypes (B1R vs. B2R) was comparable between H and T1D (*data not shown*). Similarly, mRNA levels of B1R were low in CD14^pos^ monocytes and CD34^pos^ CPC isolated from H and T1D PBMC by magnetic sorting. B2R mRNA levels were higher in CD34^pos^ CPC than in CD14^+^ monocytes, with no difference between H and T1D groups (***Fig. S1***).

### Kinin-induced migration of circulating cells

**Figure 3:**
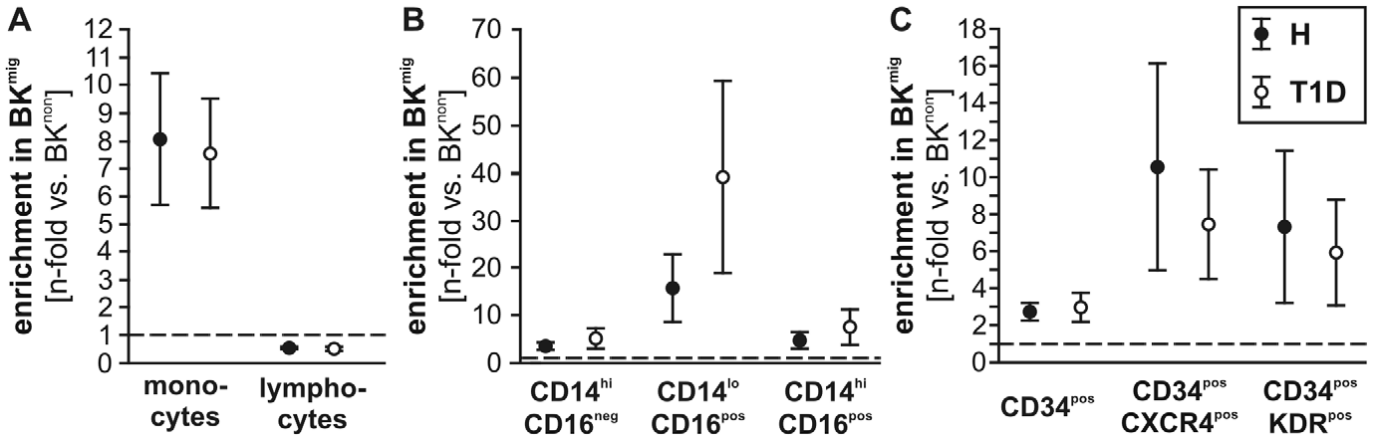
Enrichment of distinct blood cell populations by migration towards bradykinin. Monocytes (**A, B**) and CPC (**C**) were enriched in BK^mig^, as compared to BK^non^ in both donor groups, while lymphocytes were depleted (**A**). Values are mean±SEM of n = 20 (**A, C**) or n = 12 (**B**) donors per group. Dotted line indicates BK^mig^/BK^non^ ratio = 1 (i.e. no enrichment/depletion).

**Figure 4:**
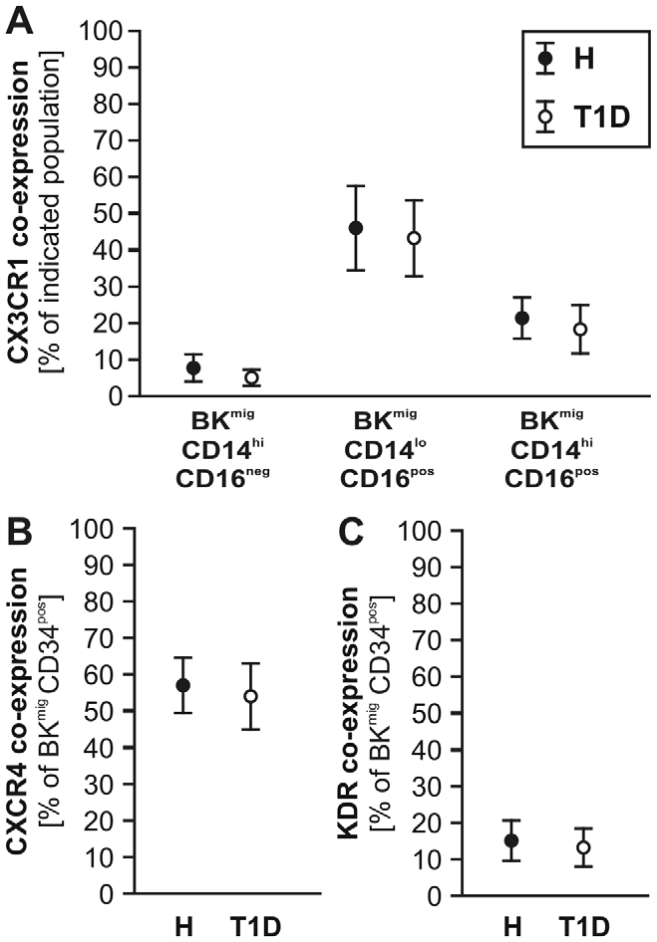
Co-expression of chemokine and growth factor receptors on migrating cells. CX3CR1 co-expression of migrating monocyte subtypes was comparable between T1D and H donors (**A**, n = 14 per group). Likewise, CXCR4 (**B**) and KDR (**C**) did not differ in their co-expression by migrating CD34^pos^ CPC between T1D and healthy subjects (n = 20 per group).

We previously reported the involvement of the B2R in CPC recruitment to ischemic tissue and subsequent promotion of reparative neovascularization [11]. Moreover, we demonstrated that by *ex vivo* migration towards BK, a cell population with enhanced pro-angiogenic characteristics (BK^mig^) can be enriched from total PB-MNC of healthy human subjects and patients with acute myocardial infarction, but not from patients with stable angina [11]. Now, comparing BK^mig^ from T1D to H, we detected an enrichment of CPC and monocyte subtypes in BK^mig^ and depletion of lymphocytes, with no significant differences between patient groups (***Fig. 3***). No significant differences in co-expression of CX3CR1, CXCR4, or KDR were detected by comparing H vs. T1D BK^mig^ monocytes or CPC (***Fig. 4***).

### Support of endothelial cell function by BK^mig^

Various mechanisms might contribute to the overall pro-angiogenic and endothelial-supportive effect seen before for healthy BK^mig^ cells and some of those could be altered in T1D patients. Both, trans-differentiation of CPC and monocytes into vascular cells - thereby replacing defect resident endothelial cells (EC) - as well as paracrine effects might play a role, with recent reports accrediting more relevance to the later [16], [17].

**Figure 5:**
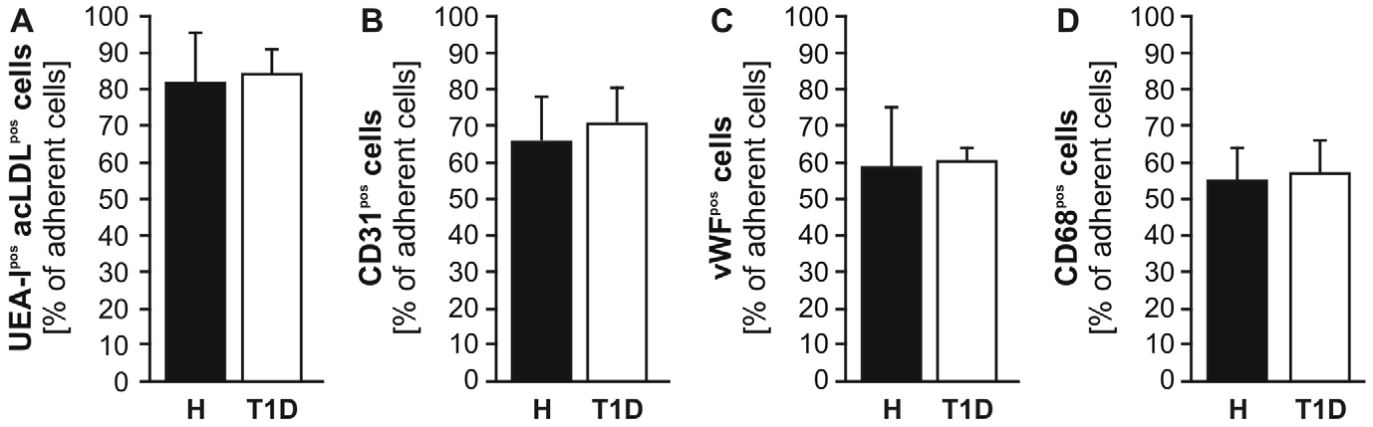
Outgrowth potential of migrating cells. BK^mig^ of both study groups gave rise to equal numbers of UEAI^pos^ acLDL^pos^ (**A**), CD31^pos^ or vWF^pos^ EC (**B and C**) or CD68^pos^ macrophages (**D**). Values are mean±SEM. n = 6 per group.

No differences between T1D- and H-derived BK^mig^ were found in their potential to give rise to acLDL^+^UEAI^+^, CD31^+^ and vWF^+^ EC or CD68^+^ macrophages in respective specific culture conditions *(* ***Fig. 5*** *)*.

**Figure 6:**
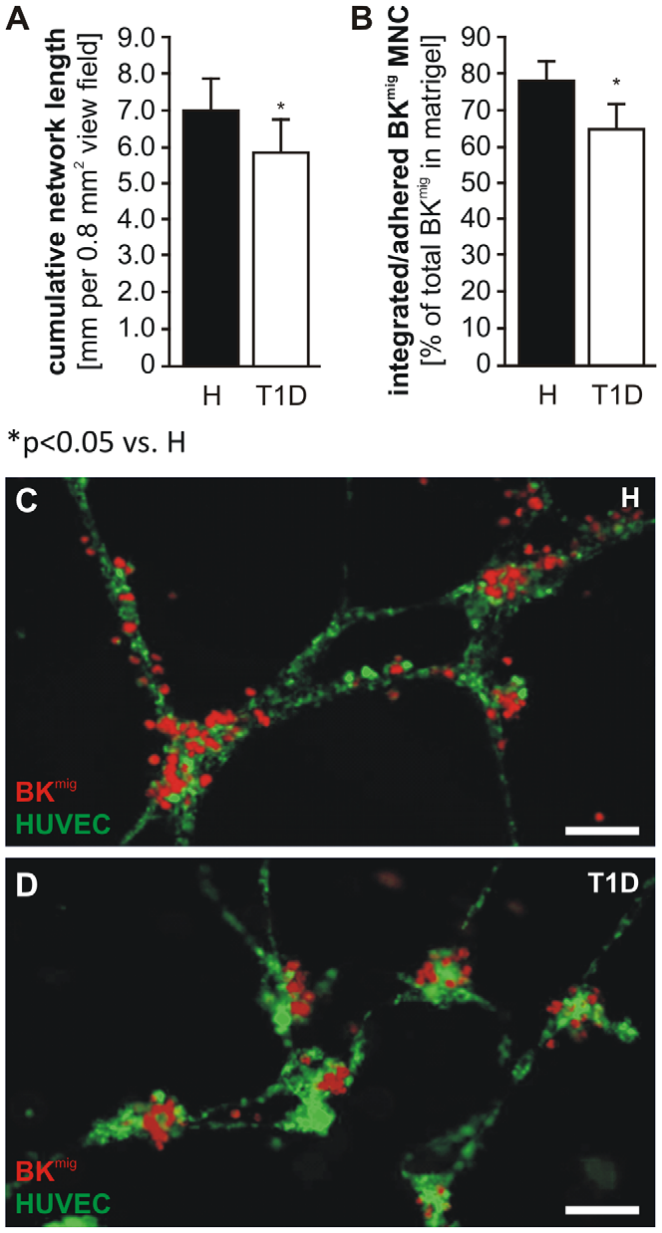
Support of endothelial network formation by migrating cells. The extension of networks (**A**) was lower if HUVEC were co-cultured with BK^mig^ from T1D donors as compared to H-derived BK^mig^. BK^mig^ MNC from H preferentially integrated to or associated with EC network structures, while a larger percentage of T1D BK^mig^ within in the gels were visible as single cells, not in contact with other cells (**B**). While T1D cells (**C**) mainly surrounded branching points, H cells (**D**) also covered branches. Values are mean±SEM. n = 10 per group, size bar equals 100µm (**C&D**) * P<0.05 vs. H.

We have previously shown that BK^mig^, but not BK^non^, support the formation of network structures by mature EC in an extracellular matrix gel [11]. Now, we observed more extensive network formation by HUVEC when they were co-cultured with H BK^mig^ as compared to T1D BK^mig^ (***Fig. 6A***). BK^mig^ MNC from H preferentially integrated to or associated with EC network structures, while a larger percentage of T1D BK^mig^ within the gels were visible as single cells, not in contact with other cells (***Fig. 6B***). Furthermore, T1D BK^mig^ were preferentially located around branching points, while H BK^mig^ also covered the branches of HUVEC networks, indicating altered inter-cellular communication of T1D cells with EC (***Fig. 6C&D***).

### Generation of NO and O_2_^−^*

**Figure 7:**
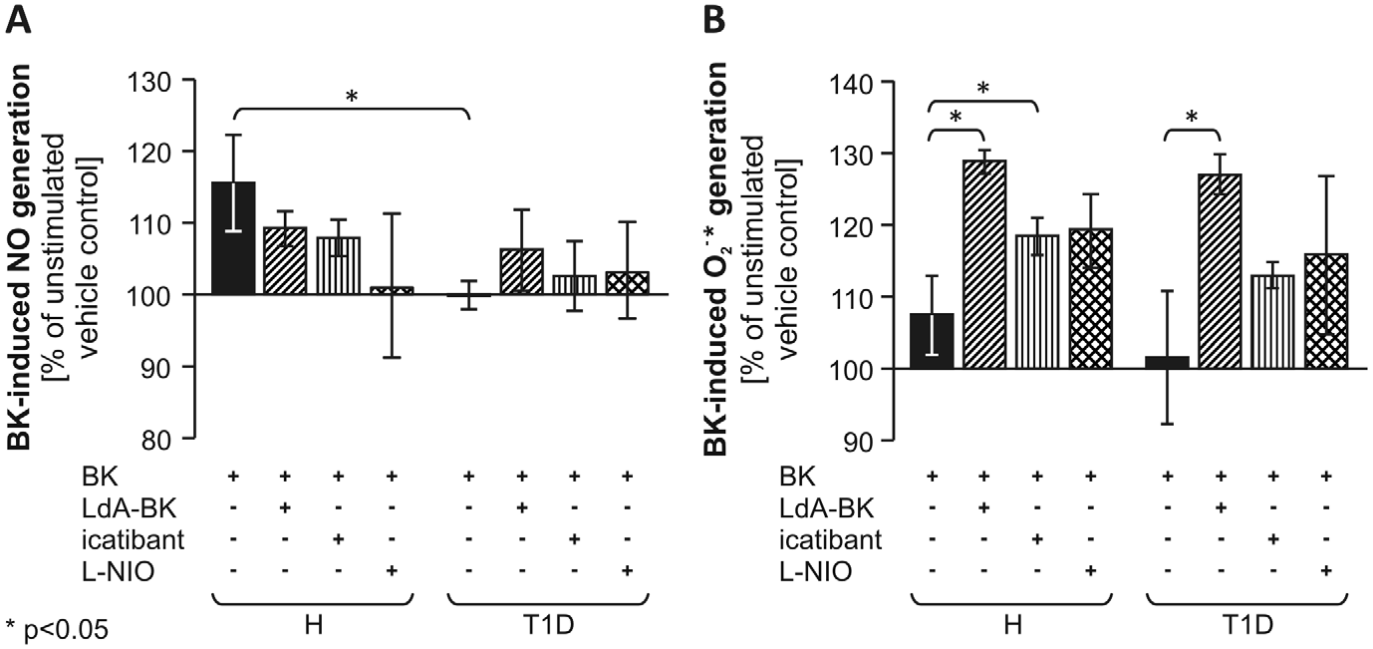
Generation of nitric oxide and superoxide by migrating cells in response to bradykinin. H BK^mig^ cells generated NO in response to BK in an eNOS dependent fashion, as indicated by inhibition by L-NIO. NO generation from T1D BK^mig^ was lower than from H cells and not inhibitable by L-NIO (**A**). Neither H nor T1D BK^mig^ generated surplus O_2_^−^* in response to BK addition (**B**). Inhibition of B1R (by LdA-BK) or B2R (by icatibant) partially inhibited NO generation (**A**) and increased O_2_^−^* generation (**B**). eNOS blockade by L-NIO enhanced O_2_^−^* levels per cell, underlining the antioxidant role of eNOS (**B**). Values are mean±SEM of n = 9 (**A**) or n = 10 (**B**) per group. * P<0.05 vs. H.

We next analyzed BK-induced NO and O_2_^−^* synthesis, whose balance is considered critical for EC survival and function. Kinins stimulate NO generation but might also induce O_2_^−^* generation by NADPH oxidase [9], [12]–[14]. BK^mig^ derived from H PBMC generated NO in response to BK stimulation through a mechanism involving both kinin receptors (being similarly blunted by B1R antagonist LdA-BK and B2R antagonist icatibant) and eNOS (being totally blocked by L-NIO), while T1D BK^mig^ did not *(* ***Fig. 7A*** *)*. In contrast, O_2_^−^* generation evoked by BK stimulation was not different between H and T1D groups *(* ***Fig. 7B*** *)*. Intriguingly, both kinin receptor antagonists increased the O_2_^−^* generation evoked by BK, suggesting the existence of a cross talk between the two receptors in the control of oxidative stress following exposure to kinin. In this respect, we could not detect any difference between H and T1D. Furthermore, BK stimulation led to elevated O_2_^−^* levels when endothelial NO synthase (eNOS) was inhibited (***Fig. 7B***), agreeing with previous reports on the anti-oxidative effects of eNOS/NO signaling [18], [19].

### Influence of differential glucose levels on CAC function

**Figure 8:**
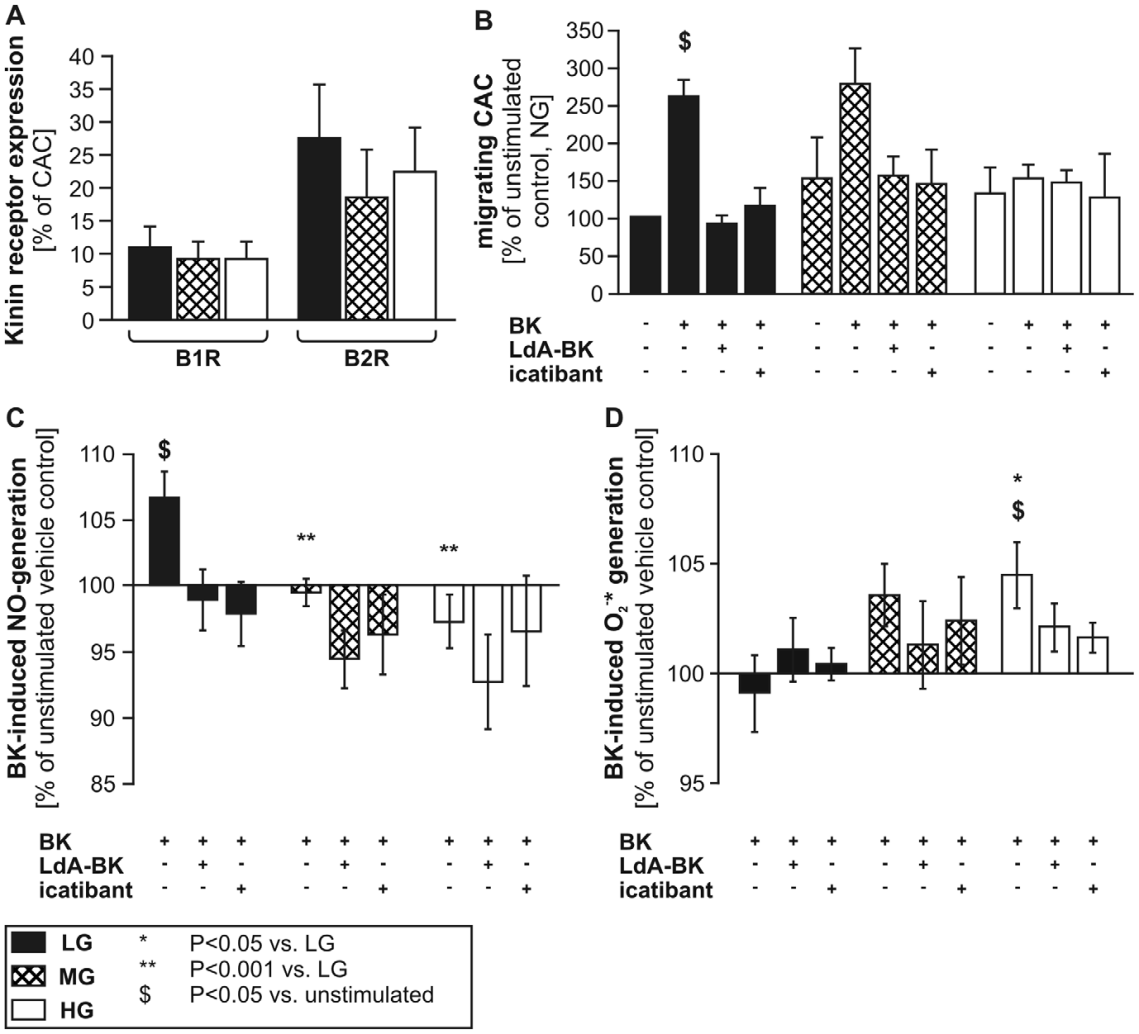
Cellular function in differential glucose conditions. Expression of B1R and B2R was not significantly different in CAC cultured under moderately increased (MG, 10mM)) or high glucose (HG, 25mM) concentrations (**A**). Migration of CAC, derived from H donors, towards BK was not affected by MG, but reduced after culture in HG (**B**). CAC cultured in low/normal glucose (LG) generated NO upon BK addition, while CAC cultured in MG or HG did not generate NO (**C**). In contrast, LG-cultured CAC did not generate O_2_^−^* upon BK stimulation, while CAC cultured in MG or HG did (**D**). Blockade of kinin receptors reduced NO generation even more in MG and HG (**C**), but also reduced O_2_^−^* production (**D**). Values are mean±SEM of n = 5 (**A**), n = 11 (**B**), or n = 9 (**C&D**) per group.

To test the hypothesis that moderately increased glucose levels (MG) influence kinin-related angiogenic cell functions differently than high glucose levels (HG), we studied the migratory activity as well as NO and O_2_^−^* generation of H CAC cultured under increasing glucose concentrations. After one week of culture in endothelial-specific medium, the majority of CAC expressed CD31 and CD11b and took up acetylated low density lipoproteins with no significant difference between glucose concentration-groups (***Fig. S2***). B1R and B2R expressions were not significantly altered in CAC under different glucose conditions (***Fig. 8A***). CAC migration towards BK was unaltered when cells were grown in low or moderately increased glucose concentrations, while CAC grown in high glucose medium migrated less (***Fig. 8B***). Addition of insulin to CAC during culture under increasing glucose conditions did not improve BK-induced migration of HG-cultured CAC (***Fig. S3***).

Culture of CAC in increasing glucose concentrations progressively reduced the cells' capacity to generate NO upon BK-stimulation and increased BK-induced O_2_^−^* generation (***Fig. 8C&D***). Different from what was observed in BK^mig^ PBMC, kinin receptor blockade only slightly increased O_2_^−^* levels in LG cultured CAC and even seemed to reduce oxidative stress in HG-cultured CAC.

## Discussion

Several types of circulating angio-supportive cells are reduced and/or dysfunctional in diabetes mellitus, including hematopoietic stem cells, endothelial progenitor cells and pro-angiogenic monocytes [1], [2]. Recent studies, addressing the question whether function and liberation of angio-supportive cells are disturbed prior to, along with, or as a result of microangiopathy, attribute high impact to the bone marrow and report dysfunctional cell liberation to underlie and precede later vascular complications [20], [21]. Besides liberation, recruitment is the other crucial step a circulating cell has to perform before exerting its effect on the target tissue. Theoretically, cell recruitment could work as another selection step, preferring still-functional cells over dysfunctional ones. In this scenario, the regenerative potential of the vessel wall would be preserved even when CPC start to get dysfunctional, as long as enough CPC are still available, able to home, and do not yet themselves contribute to endothelial dysfunction, e.g. through the exaggerated generation of oxygen radicals. Unfortunately, information about initial alterations in cell homing early in the onset of diabetes-induced vascular dysfunction is scarce, as previous studies investigated mainly the late stages of vascular disease, when CPC are dysfunctional and macrovascular damage is already evident.

So far, mechanistic research has focused mainly on SDF-1/CXCR4 signaling during the recruitment of angio-supportive cells [22]–[24]. Nevertheless, kinins play a key role in governing processes of the vessel wall and we recently demonstrated their necessity for recruiting circulating pro-angiogenic cells [8], [9], [11]. Given the relevance of BK-induced cellular functions, both during migration/cell recruitment and after migration, we aimed in the current study to analyze alterations in migratory and post-migratory activity of diabetic cells attracted by BK.

Unlike previous studies, where patients with higher HbA_1C_ and/or progressed vascular disease were tested, we compared young T1D patients with minimal microvascular disease and no clinically symptomatic macrovascular damage to age- and sex-matched non-diabetic controls. Results newly document that alterations in kinin-related post-migratory functions - but not migration itself - precede the development of diabetes-induced vasculopathy.

Two cell populations were studied in particular: CD34^pos^ CPCs and monocytes, both reportedly involved in regulation of angiogenesis and endothelial repair [25]–[27]. In contrast to our earlier study, where patients with acute myocardial infarction or stable angina showed diminished abundance of B2R on CPCs, we could not detect any significant difference in kinin-receptor expression between cells from T1D and H donors [11]. Likewise, migratory response of the studied cell types to BK was preserved in T1D patients and the similar composition of migrating cell populations was confirmed by an outgrowth assay which did not reveal any differences in the capacity of H or T1D migrating cells to give rise to EC or macrophages in culture. This result was surprising, given previous reports of migratory deficits of CD34^pos^ CPC or cultured endothelial progenitor cells, associated with impaired NO generation, and is likely to be explained by more advanced stages of vascular disease in those studies [28], [29].

Despite phenotypic similarities, BK^mig^ from H and T1D differed in their paracrine activity and support of endothelial function: T1D BK^mig^ were less potent in supporting EC network formation *in vitro* and they generated less NO than H BK^mig^.

Enhanced production of reactive oxygen species and reduced NO generation are a hallmark of diabetic EC/CPC dysfunction and considered causal for their reduced angiogenic capacity, as well as in the progression of atherosclerosis [6], [11], [30]. Surprisingly, O_2_^−^* generation by migrating T1D PB-MNC was not different from the respective H-derived cells. In our *in vitro* model, no difference in the enrichment of inflammatory monocytes or granulocytes (*data not shown*), nor in the outgrowth of monocyte-derived CD68^pos^ macrophages was detected between H and T1D PB cells, agreeing with absent changes in O_2_^−^* generation in T1D BK^mig^. Taken together, our data might shed some light on early vascular events prior to the establishment of macrovascular endothelial dysfunction: Under homeostatic conditions, EC-derived kinins support endothelial function and vascular integrity autocrinally and via the recruitment of angio-supportive cells, mainly mediated by the B2R [9]. BK-induced NO generation represents a major mechanism supporting endothelial cell function in this context, serving as a signaling molecule as well as an antioxidant [9], [18], [19]. Although BK has also been described to induce the formation of O_2_^−^* via NADPH oxidase [14], the anti-oxidant effect seems to prevail at this stage, indicated by increased levels of O_2_^−^* under kinin receptor blockade. In early stages of T1D, kinin receptor expression and migration towards BK remain unaltered in various types of circulating MNC. While BK-responsive cells still do not produce higher levels of O_2_^−^*, they lost their ability to generate sufficient levels of NO in response to BK stimulation and to support EC function. Presumably, additional deregulations in downstream molecular pathways (breakdown of oxidative defense, eNOS uncoupling) occur with additional aggravation of conditions, diverting BK-induced signaling and allowing oxygen radical production to prevail. In those conditions – like our CAC cultured under high glucose for one week – blockade of kinin receptors leads to a reduction of BK-generated oxidative stress. While future studies need to further investigate how diabetes affects intracellular processing of BK signals, our data indicate the involvement of both kinin receptors in antioxidative defense.

Patients participating in this study showed mildly elevated HbA_1c_ levels, compatible with incomplete control of hyperglycemia. Testing differentially elevated glucose concentrations in cell culture experiments, we were able to partly reproduce the *in vivo* data: Similar to the T1D patients' cells, CAC grown in moderately increased glucose (10mM) retained their migratory capacity towards BK, which was only impaired at high concentrations of glucose (25mM) during the one week culture period. Upon BK administration, CAC generated NO, but not O_2_^−^*, when cultured at normal/low glucose concentrations (5mM). Under moderately increased or high glucose concentrations, BK-induced NO generation was abrogated, and O_2_^−^* generation was induced. In addition to the phenotypic differences between CAC and BK^mig^, culture conditions might be harsher than the environment within the T1D patients, explaining that BK apparently induced pro-oxidative signaling in HG-cultured cells – which is reduced by receptor blockade – while in the T1D patients' BK^mig^ cells, kinin receptor blockade led to an increase in oxidative stress, indicating rather anti-oxidant effects of BK. Additional studies are needed to better elucidate differential signaling events initiated by moderately increased or high glucose levels as well as in early and longer persistent diabetes.

In summary, our data indicate a preservation of certain cellular functions, such as migration and low O_2_^−^* generation in well controlled young T1D patients, while NO generation and overall paracrine support of EC is already affected early on in disease onset. We therefore conclude that initially, the lack of paracrine support provided to EC by recruited cells, rather than actively inflicted damage, contributes to the loss of endothelial integrity in T1D. The increased state of inflammation, perpetuated by recruited inflammatory cells and generation of oxygen radicals, might only occur at a later stage.

## Methods

### Patient Recruitment

T1D patients (n = 28) with a background retinopathy according to the UK national screening committee for diabetic retinopathy were recruited at the Joint Clinical Research Unit of the Bristol Royal Infirmary. T1D patients with pre-proliferative and proliferative retinopathy, HbA_1C_ above 10%, or macrovascular complications (PAOD, CAD) were excluded. Respective healthy control subjects (H, n = 28) were recruited in parallel with diabetic patients to match age and gender of T1D patients in order to reduce variability. T1D and H donors with conditions reported to affect function of angiogenic cells (pregnancy, tumors, peripheral or coronary artery disease, hypertension, habitual smoking, extensive exercise training (e.g. marathon runners), over 55 years of age or taking medication affecting kinin signaling (ACE inhibitors, angiotensin receptor blockers) were excluded from both study groups. All procedures were performed in accordance with the Declaration of Helsinki and after obtaining approval of the local ethics committee (Bath Research Ethics Committee, Bath, U.K.) and written informed consent from all donors.

### Cell preparation and culture conditions

Blood was collected from matched pairs of T1D and H donors, anonymized by the study nurse and processed in parallel by a researcher blinded for group affiliation. Peripheral blood mononuclear cells (PB-MNC) were prepared from fresh EDTA-anticoagulated blood within 1 hour after withdrawal using density gradient centrifugation, as before [11]. Cells migrating towards BK (BK^mig^) and the respective non-migrating cells (BK^non^) were derived from PB-MNC as described previously [11]. Cultured angiogenic cells (CAC) were obtained from PB-MNC by culture in endothelial-specific medium (EGM-2 MV, 10% bovine serum) for 5 days as previously described [11]. For some experiments, CAC of H donors were cultured in normal/low (LG, 5mM), medium (MG, 10mM), and high (HG, 25mM) glucose medium. Recombinant human insulin (1U/mL) was added to duplicate LG, MG and HG groups during the culture period in some experiments.

### Antigenic characterization

Individual cell populations in PB, BK^mig^ and BK^non^ were identified according to their surface expression levels of CD34 (Miltenyi), KDR (R&D systems), CXCR4 (BD), CD14 (Miltenyi), CD16 (BD), CX3CR1 (Caltag) by flow cytometry (***Fig. S4***). Unlabelled primary antibodies against kinin B1 (B1R) and B2 receptor (B2R) were revealed by FITC-labeled anti-rabbit antibody (all Sigma). Isotype and secondary antibody controls were performed for each staining to verify specificity. 2×10^5^ to 3×10^5^ total events were acquired on a FACSCanto II flow cytometer and analyzed with FACSDiva 6.1.2 software (both BD). Enrichment or depletion of distinct cell types within BK^mig^ was calculated versus BK^non^ of the same experiment as before [11]. Expression of endothelial cell (von Willebrand Factor, CD31) and macrophage antigens (CD68), uptake of acetylated low-density lipoproteins (acLDL) and binding of *ulex europaeus* agglutinin I (UEAI) were studied by immunofluorescence microscopy in adherent BK^mig^-derived cells cultured under cell-type specific conditions.

### Quantitative RT-PCR

CD14^pos^ monocytes were isolated by magnetic sorting following the manufacturers recommendations (Miltenyi). Cells were enriched using 2 successive columns to increase purity. CD14^neg^ cells were labeled with anti-CD34 magnetic beads (Miltenyi) and CD34^pos^CD14^neg^ CPC isolated likewise. 10^5^ isolated cells of each population were lysed and reverse transcribed using the Power SYBR® Green Cells-to-Ct™ kit (Ambion). cDNA levels of B1R and B2R, as well as 18S rRNA were quantified by qPCR using the same kit and a DNA Engine Opticon 2 (BioRad).

### Functional cell characterization

The ability of BK^mig^ to support network formation of human umbilical vein EC (HUVEC) was studied in a matrigel assay as reported previously [11]. Generation of nitric oxide (NO) and superoxide (O_2_^−^*) was assessed using the fluorescent probes diaminofluorescein (DAF-2DA) and dihydroethidium (DHE, both invitrogen), respectively, by fluorescence microscopy or analysis by the IN Cell Analyzer (GE Healthcare). Briefly, nuclei were labeled with 5nM Hoechst 33342 (Sigma), and cells pre-incubated with either antagonists of the B1R (Lys-(des-Arg9, Leu8)-Bradykinin, LdA-BK, Sigma, final concentration 0.5 µM), the B2R (icatibant, Sigma, final concentration 0.5 µM), or eNOS (L-NIO, 1mM). DAF or DHE (final concentration 5µM) and BK (final concentration 0.1µM) were added. Controls contained no stimulus, but the respective fluorescent probe. Fluorescence intensity was measured using an IN Cell Analyzer 1000 (GE Healthcare, Amersham, UK). Images were acquired using a 10× objective, q505LP dichroic mirror (Chroma Technology Corp, Rockingham, VT) and 360-40nm (Hoechst) or 475-20nm (DHE /DAF) excitation filters with 460-40nm, 570-30nm (DHE) or 535-50nm (DAF) emission filters. Three independent experiments were performed in triplicate wells each with two fields of view (each 0.6mm^2^) per well. Fluorescence intensity (FI) was assessed, initially at baseline (0 min) then after 15min for O_2_^−^* measurement or after 1h and 3h for NO measurement. Image analysis was performed using IN Cell Analyzer Workstation 3.5 software (IN Cell Investigator, GE Healthcare) and a Dual Area Object Analysis algorithm as previously described [31]. Only cells with a cytoplasmic FI greater than 10% of background intensity were considered. Imaging data is reported as the mean cytoplasmic FI per cell.

Similarly, BK-induced NO and O_2_^−^* generation by BK^mig^ or BK^non^ of H and T1D was studied by using fluorescence microscopy. Vehicle controls contained DAF or DHE, but no inhibitor or BK. Experiments were performed in duplicate for each patient. Fluorescence microscopic images were taken after 15min (DHE, red filter) and 1h (DAF, green filter), together with Hoechst-stained nuclei (blue filter) of the same viewfield in a Zeiss microscope/camera system with fixed exposure settings. Fluorescence intensity per cell was then assessed using ImageJ v. 1.41 (NIH). Mean FI per cell is reported.

### Statistical analysis

Normally distributed values were compared by paired t-test (two groups) or repeated measures ANOVA (multiple groups, followed by Holm-Sidak *post hoc* test). Not normally distributed values were compared by Mann-Whitney U test (two groups) or ANOVA on Ranks (multiple groups, followed by Dunn's *post hoc* test). A p-value below 0.05 was considered significant.

## Supporting Information

Figure S1 mRNA levels of B1R and B2R in magnetically isolated CD14^pos^ monocytes and CD34^pos^CD14^neg^ CPC. Values are mean ± S.E.M. of n = 4 values.
(0.07 MB JPG)

Figure S2 Expression of CD11b (B&E) and CD31 (C&F), as well as uptake of DiI-labelled acetylated low density lipoproteins (D&G) by CAC was not altered by glucose concentration in the medium. A: typical FSC/SSC plot of CAC at day 5 of culture. Values are mean ± S.E.M. of n = 4 values.
(0.35 MB JPG)

Figure S3 Impaired migration of CAC cultured under high glucose was not rescued by additional presence of insulin. Values are mean ± S.E.M. of n = 4 values.
(0.16 MB JPG)

Figure S4 Lymphocytes (ly), monocytes (mo) and granulocytes (gra) were identified based on their light scatter characteristics (A). Gates were set up for each fluorophore and antibody separately, using secondary antibody (B) and isotype controls (E, G, I & L). Representative examples show analysis of kinin B1 and B2 receptor expression (C&D), CD14^hi^CD16^neg^ (M1), CD14^hi^CD16^pos^ (M2) and CD14^lo^CD16^pos^ (M3) monocyte subpopulations (F), CD34^pos^ (H), CXCR4 (K) and KDR (M). Co-expression was analyzed by logically combining gates for fluorophores with lympho- and monocytes the analysis, e.g. CD34^pos^ CPC = (mo OR ly) AND CD34^pos^, CD14^hi^CD16^neg^ monocytes = mo AND M1 during.
(0.71 MB JPG)
