# Supplementary figures and images for: Distinct Kinin-Induced Functions Are Altered in Circulating Cells of Young Type 1 Diabetic Patients
Source: PLoS One. 2010 Jun 17;5(6):e11146. doi: 10.1371/journal.pone.0011146 (PMC2887352; doi:10.1371/journal.pone.0011146)

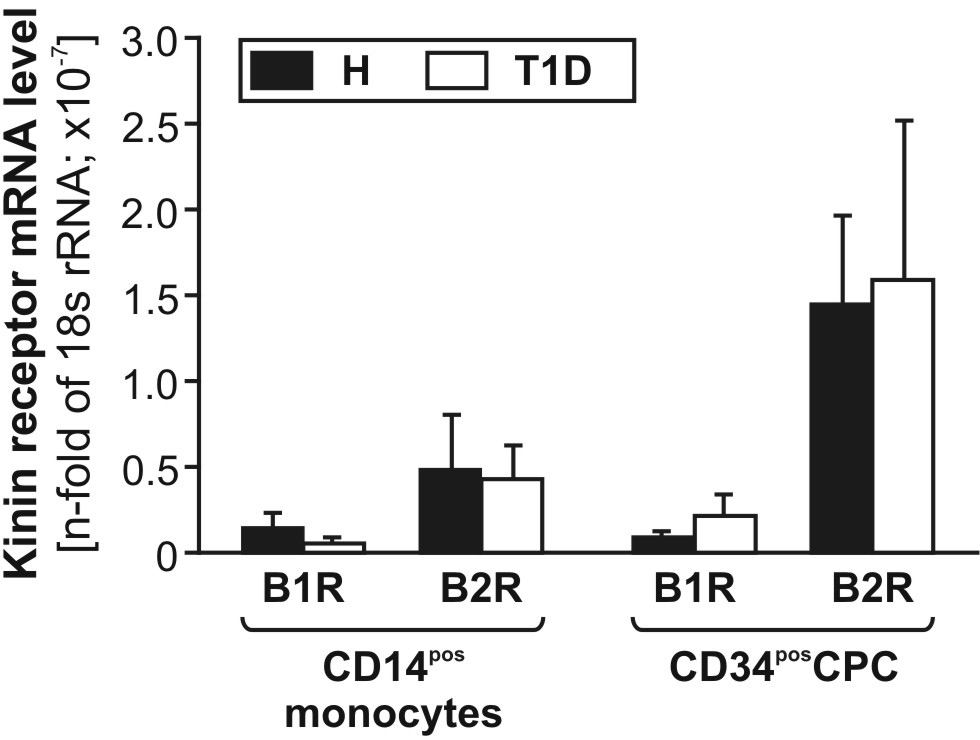

Supplement: Figure S1 — mRNA levels of B1R and B2R in magnetically isolated CD14pos monocytes and CD34posCD14neg CPC. Values are mean ± S.E.M. of n = 4 values. (0.07 MB JPG) [file pone.0011146.s001.jpg]

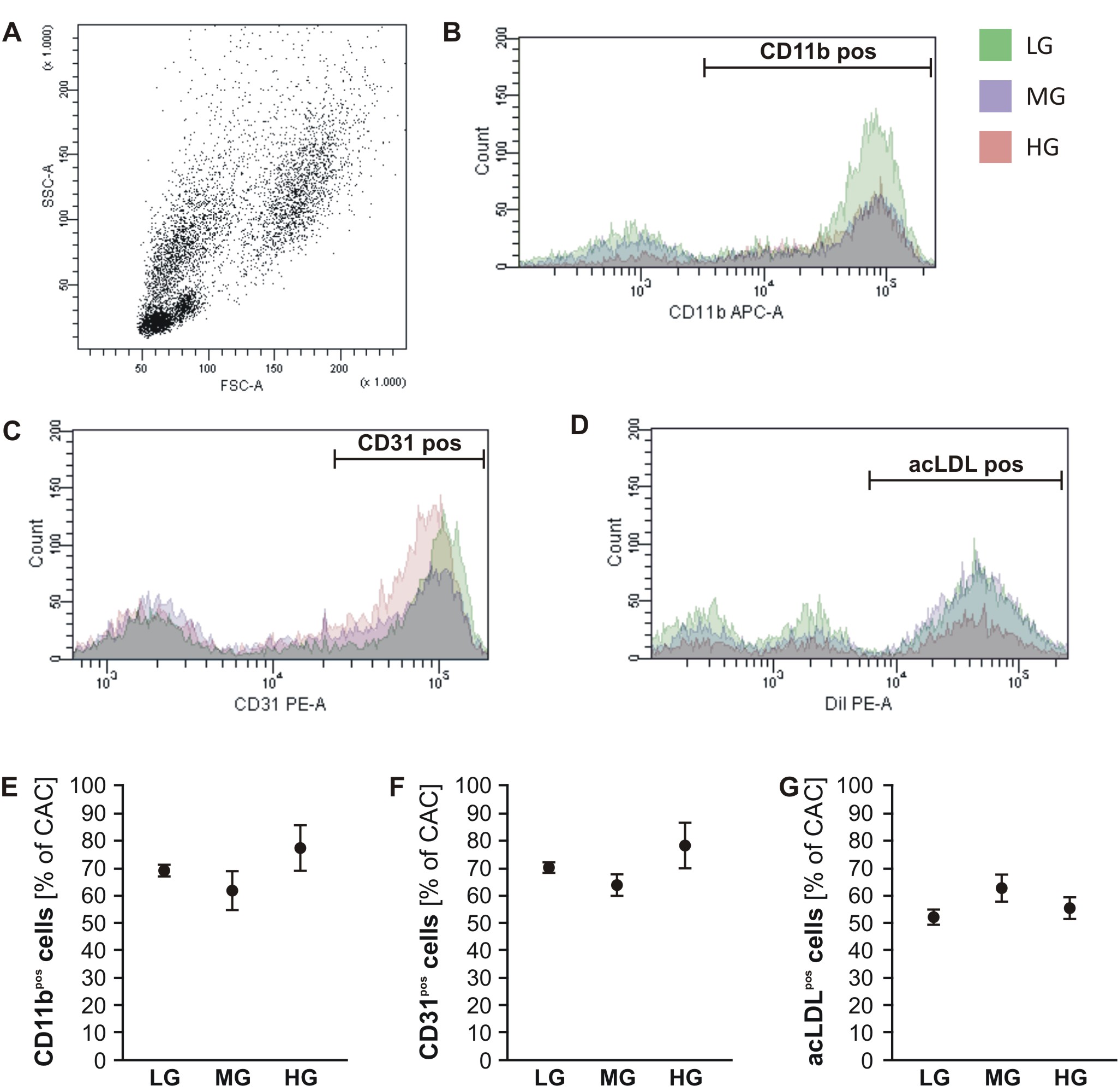

Supplement: Figure S2 — Expression of CD11b (B&E) and CD31 (C&F), as well as uptake of DiI-labelled acetylated low density lipoproteins (D&G) by CAC was not altered by glucose concentration in the medium. A: typical FSC/SSC plot of CAC at day 5 of culture. Values are mean ± S.E.M. of n = 4 values. (0.35 MB JPG) [file pone.0011146.s002.jpg]

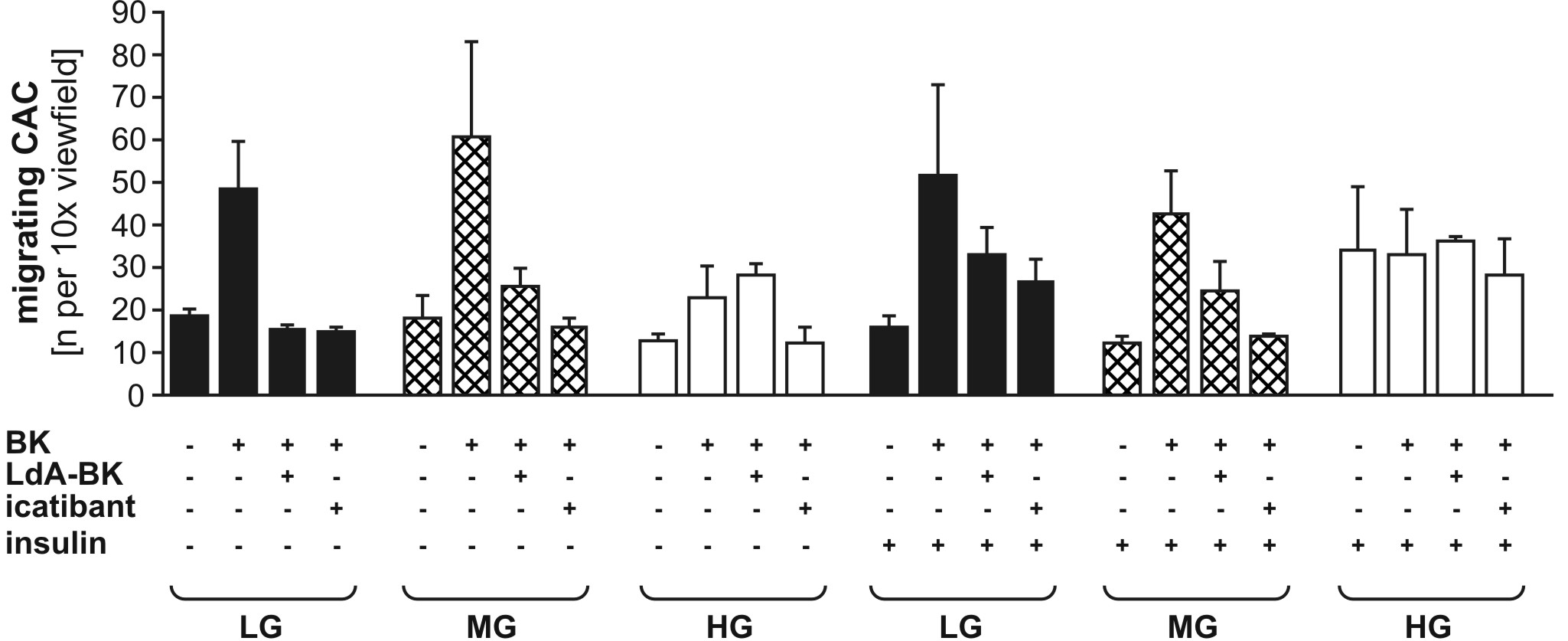

Supplement: Figure S3 — Impaired migration of CAC cultured under high glucose was not rescued by additional presence of insulin. Values are mean ± S.E.M. of n = 4 values. (0.16 MB JPG) [file pone.0011146.s003.jpg]

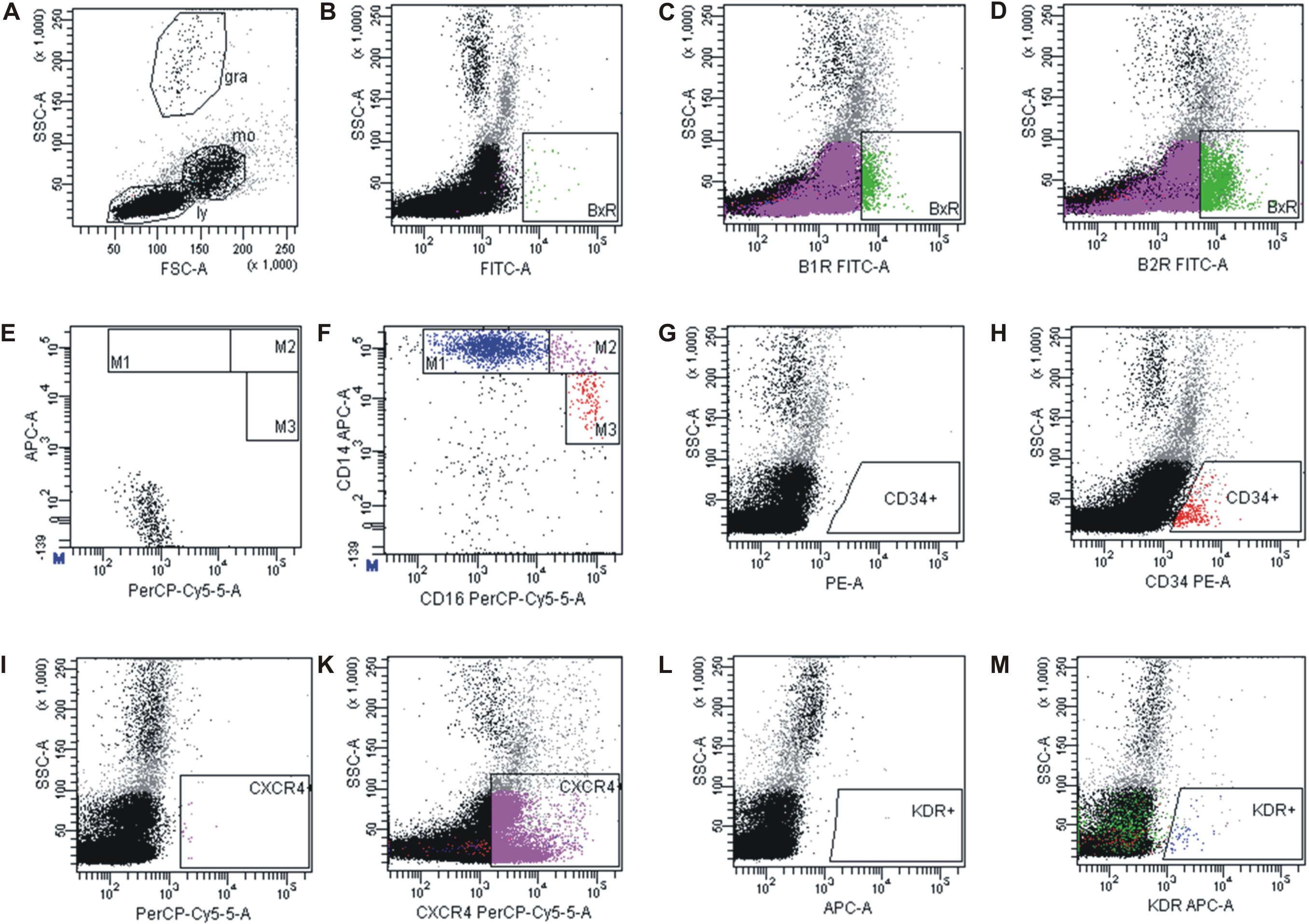

Supplement: Figure S4 — Lymphocytes (ly), monocytes (mo) and granulocytes (gra) were identified based on their light scatter characteristics (A). Gates were set up for each fluorophore and antibody separately, using secondary antibody (B) and isotype controls (E, G, I & L). Representative examples show analysis of kinin B1 and B2 receptor expression (C&D), CD14hiCD16neg (M1), CD14hiCD16pos (M2) and CD14loCD16pos (M3) monocyte subpopulations (F), CD34pos (H), CXCR4 (K) and KDR (M). Co-expression was analyzed by logically combining gates for fluorophores with lympho- and monocytes the analysis, e.g. CD34pos CPC = (mo OR ly) AND CD34pos, CD14hiCD16neg monocytes = mo AND M1 during. (0.71 MB JPG) [file pone.0011146.s004.jpg]
